# P-1578. Designing a collaboration tool for equitable international HIV research: the Uganda-US experience

**DOI:** 10.1093/ofid/ofaf695.1757

**Published:** 2026-01-11

**Authors:** Chelsea Modlin, Harriet Nankya, Larry Chang, Joseph Ali, Nelson Sewankambo

**Affiliations:** Johns Hopkins University School of Medicine, Baltimore, MD; Makerere College of Health Sciences, Kampala, Kampala, Uganda; Johns Hopkins School of Medicine, Baltimore, Maryland; Johns Hopkins Bloomberg School of Public Health, Baltimore, Maryland; Makerere College of Health Sciences, Kampala, Kampala, Uganda

## Abstract

**Background:**

Prioritizing mutually beneficial inputs, processes, outputs and impact between partners within international research partnerships (IRPs) is an ethical imperative to achieve meaningful scientific aims. The goal of this project is to capture themes and topics from the Uganda-US HIV IRP experience to include in the design of a tool to assist partnerships in addressing research inequities.

**Figure 1.**
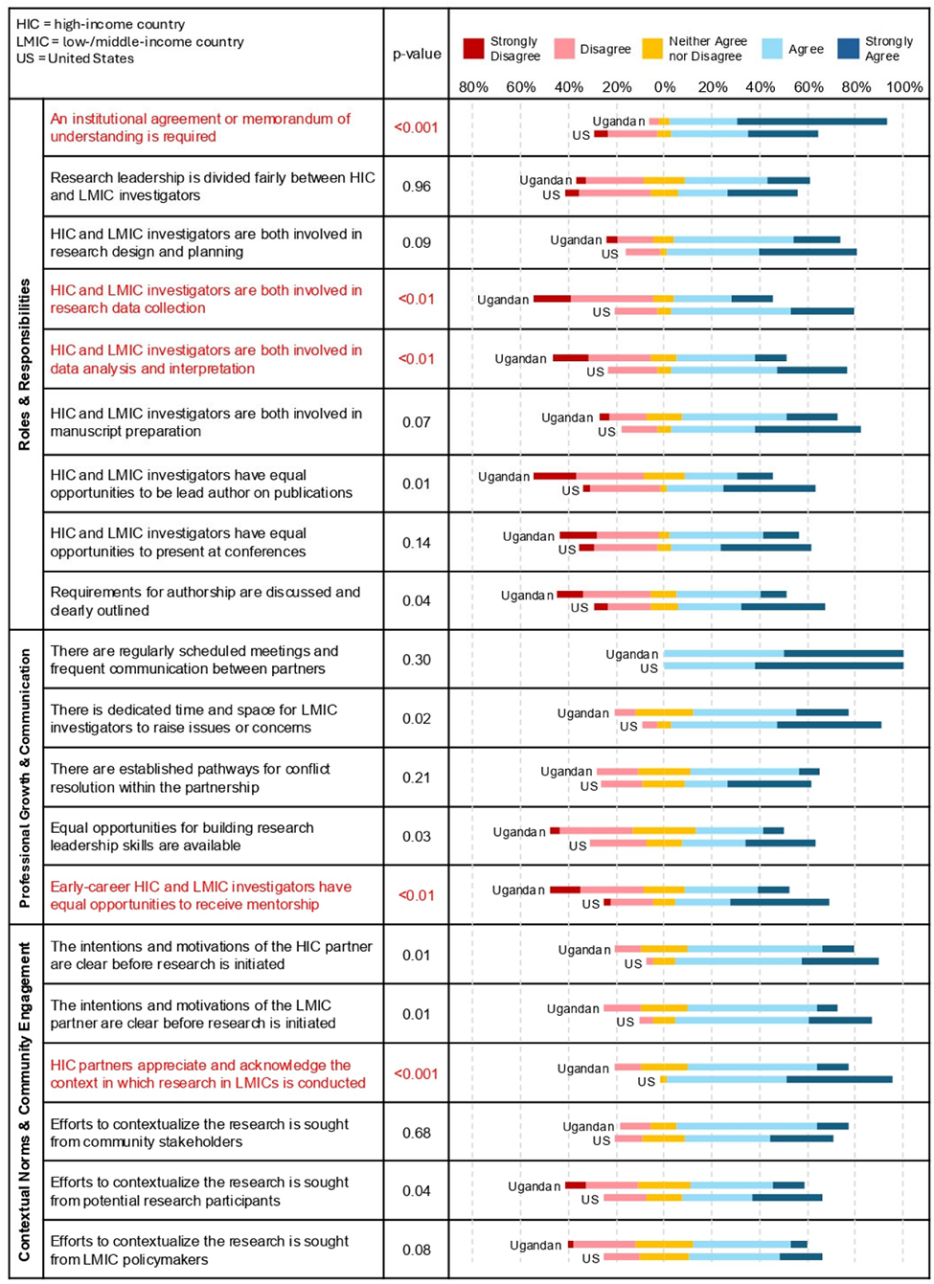
Example of survey output demonstrating differences between Ugandan and US respondents. Answers to questions were six single-choice Likert responses ranging from 'Strongly Agree' to 'Strongly Disagree' with one neutral response and one 'unknown/unsure' response option.

**Methods:**

Exploratory quantitative (survey) to qualitative (in depth interviews) mixed-method design among Ugandan and US HIV researchers and research staff. Survey results were analyzed using descriptive statistics. Interview transcripts were analyzed using exploratory and confirmatory thematic analysis.

**Results:**

Eighty surveys and 16 interviews took place November 2024-April 2025. The figure demonstrates an example of survey output. Disagreement between Ugandan and US researchers involved the inclusion of less advantaged researchers and communities in priority setting, data analysis, and scientific interpretation, accessing academic mentorship, and a lack of US researcher appreciation for the context in which research in Uganda takes place. Responsibility for monitoring and improving these outcomes was distributed between researchers, research institutions, and research funders. Areas of agreement between Ugandan and US researchers included a highly developed scientific capacity within Uganda, funded most often through international efforts, but a lack of support for the administrative and infrastructure capacity needed to reach scientific independence. Accountability for this lack of development was attributed to policies within institutions and funders.

**Conclusion:**

IRP processes identified as inequitable are often rooted in larger global health systems such as the influence funders and limitations around institutional and national scientific infrastructure. While these are challenging for researchers or IRPs to address, areas of discordance between Ugandan and US researchers, such as setting the research agenda, developing the science within a more informed understanding of Ugandan contexts, and increasing accessibility of mentorship and data analysis skills may be optimal targets for developing a collaboration tool for international research partnerships.

**Disclosures:**

All Authors: No reported disclosures

